# Model selection and parameter estimation for root architecture models using likelihood-free inference

**DOI:** 10.1098/rsif.2019.0293

**Published:** 2019-07-10

**Authors:** Clare Ziegler, Rosemary J. Dyson, Iain G. Johnston

**Affiliations:** 1 School of Biosciences, University of Birmingham, Birmingham, UK; 2 Birmingham Institute of Forest Research, University of Birmingham, Birmingham, UK; 3 School of Mathematics, University of Birmingham, Birmingham, UK; 4 Alan Turing Institute, London, UK; 5 Faculty of Mathematics and Natural Sciences, University of Bergen, Bergen, Norway

**Keywords:** likelihood-free inference, root system architecture, root growth, approximate Bayesian computation

## Abstract

Plant root systems play vital roles in the biosphere, environment and agriculture, but the quantitative principles governing their growth and architecture remain poorly understood. The ‘forward problem’ of what root forms can arise from given models and parameters has been well studied through modelling and simulation, but comparatively little attention has been given to the ‘inverse problem’: what models and parameters are responsible for producing an experimentally observed root system? Here, we propose the use of approximate Bayesian computation (ABC) to infer mechanistic parameters governing root growth and architecture, allowing us to learn and quantify uncertainty in parameters and model structures using observed root architectures. We demonstrate the use of this platform on synthetic and experimental root data and show how it may be used to identify growth mechanisms and characterize growth parameters in different mutants. Our highly adaptable framework can be used to gain mechanistic insight into the generation of observed root system architectures.

## 1. Introduction

Root systems are essential to plants’ structure and uptake of water and nutrients and constitute more than 5% by mass of the total global carbon budget [1]. They stabilize plants [2], stabilize soils [3], foster beneficial microbes [4] and are the entry point for water and nutrients to the plant [5]. The shape of a plant’s root system is generated by a variety of physiological and signalling pathways within the plant, and understanding the generation of this system opens paths to its optimization to maximize crop yield [6].

Despite this importance, the mechanisms underlying root growth remain challenging to quantitatively understand [7–9]. The complexity of root systems and their below-ground nature poses observational challenges. Experimental techniques aiming to elucidate root architecture have historically included sketches of root systems and the use of hydroponics, then images of cleaned root systems. More recent advances have facilitated the imaging of plants *in situ* through the use of X-ray *µ*-computed tomography [10], magnetic resonance imaging scanning [11,12] and transparent soil [13], which have been used to investigate root soil exploration and uptake of water and nutrients.

In parallel with this experimental elucidation, in an effort to understand how root systems grow, many physical and mathematical models of root growth and structure have been produced. These models attempt to solve the ‘forward problem’: given knowledge of the parameters governing growth processes in plants, they produce the details and dynamics of a likely simulated root system. For example, the Lockhart equation described the elongation of a cell under turgor pressure [14] and has been widely adapted to describe the growth of many plant organs, including roots [15,16]. Hackett & Rose produced the first root system model in the 1970s [17] based on the growth and branching of barley roots, while Lungley [18] produced a computational model which generated root systems represented using ASCII characters. Fitter [19] introduced a topological model of root architecture where a root system was considered as a set of links. This idea was extended in the three-dimensional modelling of Pagès *et al*. [20] and Diggle, whose ROOTMAP model could be applied to a variety of plant species [21], while Tatsumi *et al.* represented variation in root systems using fractal analysis [22,23]. Lynch *et al.* modelled a root system as a network with nodes as branches and inter-branch distances as edges. The model also included root radius and volume changes along the growing root [24]. Advances in computation have led to a further plethora of root system architecture models, which produce a three-dimensional reproduction of a root system using a detailed parameter set [7]. Previous modelling approaches were combined in the production of Root Typ in 2004 [25]. This has allowed the underlying model structure to be adapted for use by other researchers [26,27]. Another key root architecture model is RootBox [28], which is designed to be combined with soil and water uptake models along with allowing for the simulation of roots grown in containers of user-defined shape and dimensions. This model has been recently updated to produce CRootBox, and there are plans to eventually extend the modelling approach to also consider above-ground plant growth [29]. Many of these effective models are able to reproduce root systems of many different plant species, which necessitates the incorporation of root data collected *in situ* [8].

While a great number of advances have been made in simulating root systems from a set of parameters, relatively little work has been done on the inverse problem: extracting the growth parameters from an observed root system. Model parametrization is often limited by difficulties in root observation [27,30]; however, this step is crucial to gain biological insight from these root models. It is vital that we can validate root models and the predictions they make, and quantify uncertainty in their mechanisms and parameters. A manual approach to the inverse problem, feeding specific measured parameters into generative models for root systems and assessing their ability to reproduce observations, has been used to gain biological insight and validate generative models [31]. However, without an automated approach, it remains challenging to explore the full ranges of parameters and mechanisms that could give rise to observed structures and the likelihoods of each. Advancing technologies are allowing observation of root systems in increasing detail, making it even more important to bridge the gap between theory and observation.

A major challenge in solving the inverse problem with traditional statistical methods is finding a likelihood function for an observed root system. Modern statistical approaches allow this challenge to be circumvented through the use of stochastic simulation and approximate Bayesian computation (ABC) techniques [32], which produce a computational approximation replacing the likelihood and remove the need for its explicit calculation. Another strength of these techniques lies in their natural capacity for model selection and the inclusion of prior knowledge about the system in an inference setting. Here, we report a novel pipeline by which ABC, embedded in a sequential Monte Carlo (SMC) framework [33], can be used to learn the values of, and uncertainty in, generative, mechanistic parameters underlying root growth and architecture, and to compare different root architecture models. *Arabidopsis thaliana* (thale cress) is used in both computational and experimental investigation throughout as a model plant, but this process can readily be extended to any root system, as we also demonstrate with *Lupinus angustifolius* (narrowleaf lupin). We demonstrate how this framework can be used to identify generative parameters according to a given model, distinguish phenotypic differences, and evaluate the comparative effectiveness of different models for root elongation and root branching processes, providing insight into the underlying mechanisms.

## 2. Results

### 2.1. An ABC SMC framework for inferring mechanistic parameters from root systems

For generality, we begin by considering a highly simplified model for root growth (see Material and methods). Starting from an infinitesimal initial condition, a primary root elongates according to a growth law. Branches from this primary root occur stochastically according to a branching law. Branches elongate according to the primary root growth law multiplicatively scaled (allowing, for example, branches to grow at a slower rate than the primary root). Branching is for now restricted to first-order branches from the primary root, though nothing in our framework is dependent on this or any other structural choice.

This coarse-grained model was chosen to reflect the core behaviour shared at the intersection of several contemporary root models [25,28,29]. Its computational simplicity is an advantage but not a necessity for our inference framework; we later consider an alternative generative model to demonstrate the transferability of our approach. The details of the model are described in Material and methods, but in this section we consider constructing the inference framework for a general mechanistic model, the parameters of which we denote *θ*.

The platform proceeds by simulating outputs from this model with different trial parametrizations, using a distance function to compare these outputs to summary statistics of experimental observations, and iterating this process within a Bayesian framework to build up posterior distributions on model structures and parameters given the observed data.

To compare simulation to experiment, we focus on a mechanistically informative set of summary statistics. For a given observation of root structure data *d*, these are the number of branches *B*, length of the primary root *L* and average length of the lateral roots $\hat{l}$. Within our scheme, these lengths are for convenience measured in centimetres; different scalings of these features can be used to emphasize different aspects of root architecture in the simulation-data comparison. The distance function we use to compare two structures *d*_1_, *d*_2_ is based on the Euclidean distance

2.1 $$\rho (d_{1},d_{2})=\frac{1}{3}({\left(B_{1}-B_{2}\right)}^{2}+{\left(L_{1}-L_{2}\right)}^{2}+{\left({\hat{l}}_{1}-{\hat{l}}_{2}\right)}^{2}).$$

A dataset D may consist of a set of structures *d*_*ij*_, where *i* labels individual plants and *j* labels longitudinal observations. In this case, for each observed plant *i*, a model plant is simulated and its structure recorded at each of the times corresponding to the longitudinal observations. We will call these recorded structures *d*′_*ij*_ and are interested in the comparison between each recorded structure and its observed counterpart

2.2 $$\rho (D,D^{\prime })=\sum_{i}\sum_{\;j|i}\rho (d_{ij},d_{ij}^{\prime }).$$

We deliberately choose this model and summary statistics to focus on the topological aspects of root architecture and ignore any specific physical embeddings (for example, branching angles). This focus on topological degrees of freedom increases the generality of the approach, but features like branching angles and higher-order topological statistics can readily be included in the modelling and distance calculation if they reflect important degrees of freedom for the scientific question under consideration.

Equation (2.1) balances the ability to capture the fine detail of the root system against the computational time required to obtain a reasonable number of samples from the posterior. Including more detail and/or degrees of freedom in the distance function will allow more detailed matching of observations but will increase the sampling effort required to find regions of parameter space that match these criteria.

ABC involves accepting a trial set of parameters as a sample from the posterior distribution when $\rho (D,D^{\prime })<\epsilon$, or, in other words, when the summary statistics of the structure emerging from the simulation are ‘close’ to those arising from the experimental data. The posterior distribution on parameters *θ* built up from a set of samples taken in this way is P(θ|D;ρ<ϵ), which forms an increasingly good approximation to the true posterior P(θ|D) as *ε* is decreased [34].

For parametric inference within a fixed model, a simple rejection-sampling pipeline is then given by algorithm 1 (Material and methods). This approach would be sufficient to identify generative parameters from data, but rejection sampling is an inefficient paradigm, as any ‘good’ regions of parameter space are immediately forgotten when the next draw from the prior is made. To facilitate more efficient parametric inference as well as model selection, we use ABC embedded in a SMC framework as in Toni *et al.* [33]. ABC SMC first enforces only a relaxed fit to the data then sequentially uses the inferred parameter distributions as effective priors while enforcing a tighter fit to data. This sequential process is parametrized by a sequence of *ε* values describing the fit threshold required at each step in the sequence. Model selection can proceed by including a ‘model index’ parameter describing which model structure is to be used, applying a prior to this parameter (thus incorporating prior knowledge about which model structures are more likely), then treating this index as a parameter to be inferred through SMC. Following Toni *et al.* [33], the coupled inference and model selection pipeline is then given by algorithm 2 (Material and methods).

### 2.2. Inferring parameters from a simulated root system

**Figure 1.**
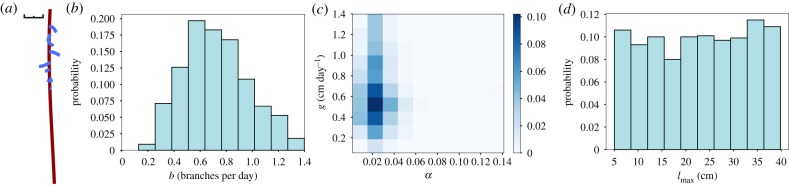
Validating root-inference platform with synthetic *Arabidopsis* data. (*a*) Output from CRootBox simulation of *Arabidopsis* root growth; black scale bar is 1 cm. (*b*–*d*) Output posteriors from an ABC SMC framework run on CRootBox output, with final ABC SMC tolerance *ε* = 0.5 (Material and methods). (*b*) Posterior distribution on branching rate *b* in the growth model. (*c*) Two-dimensional posterior on primary root growth rate *g* and lateral root growth scaling *α*. (*d*) Posterior distribution on *l*_max_, the maximum length parameter in the negative-exponential growth model used.

We first sought to test the applicability of our likelihood-free inference process on synthetic root data, to confirm its ability to identify known generative parameters. To this end, the CRootBox root simulation model [29] was used to produce an example of an *Arabidopsis thaliana* root system. The governing parameters were mean growth rates of 0.49 cm day^−1^ for the primary root, 0.08 cm day^−1^ for the lateral roots, and an inter-lateral distance of 0.2 cm, although the inter-lateral distance is not an explicit parameter in our model (see next section). CRootBox adds an element of stochasticity to its generative parameters; in the default *Arabidopsis* case, this corresponded to a coefficient of variation of 0.1 in the growth rates and 0.45 in the lateral spacing parameters. The simulation was run over 15 simulated days, yielding the structure in figure 1*a*.

To mirror the pipeline that will be used for experimental data, we analysed this simulation output with SmartRoot image analysis software [35], obtaining the statistics of tap and lateral root length and placement. We then applied our ABC SMC framework to estimate posterior distributions on the mechanistic parameters of our simple growth model (Material and methods). These parameters are *g* (primary root growth rate), *l*_max_ (primary root scaling constant), *b* (branching rate) and *α* (lateral root growth scaling).

As shown in figure 1*b*–*d*, the growth rates and, notably, their variability are well captured in the resultant posteriors, with *g* inferred to lie around 0.55 ± 0.10 cm day^−1^, compatible with the true growth rate parametrizing the synthetic data. The branching rate parameter is more broadly spread, with a mean of 0.6 day^−1^ corresponding to the observed number of branches, and flexibility in the posterior reflecting the stochastic nature of this parameter’s influence. *α* was inferred to lie around 0.021 ± 0.02, corresponding to a lateral growth rate around $0-0.02\;{\text{cm day}}^{-1}$; this is rather lower than the value used in the simulation, reflecting the rather limited lateral growth occurring in the specific simulated instance of the model. The posterior for *l*_max_ is close to recovering the prior, which suggests that the model output is minimally dependent on the value of this parameter. We found this limited *l*_max_ dependence to generally be the case, and in subsequent sections will omit *l*_max_ from the posterior plots; all *l*_max_ posteriors, generally recovering priors, are plotted in the electronic supplementary material, figure S1. This assessment of the relative importance of, and flexibility in, generative mechanistic parameters reflects a powerful aspect of this inverse modelling approach.

### 2.3. Inferring mechanistic parameters for other synthetic phenotypes and root simulation models

To test the wider applicability of our likelihood-free inference process, we next tested the ability to identify known generative parameters when using a different, existing root simulation model, and for different plant species. RootBox [28] was chosen for its wide application in the field. We embedded RootBox as the generative model in our inference framework, which was then applied as in §2.2 to the previous synthetic *Arabidopsis thaliana* data and a simulated *Lupinus angustifolius* root system. The *Lupinus* simulation involved an initial growth rate of 1 cm day^−1^ for the primary root, 0.2 cm day^−1^ for the laterals and an inter-root distance of 0.9 cm, and proceeded for 15 simulation days.

RootBox employs a different branching protocol from our simple model above. Rather than allowing stochastic branching anywhere on the primary root, RootBox allows lateral branches to emerge at specified intervals along the primary structure. This interval *d*, and a value *b*_max_ governing the maximum number of allowed lateral branches, are parameters of the model and we therefore seek posterior distributions on these quantities as well as the other mechanistic parameters which directly map to those in our simple model.

**Figure 2.**
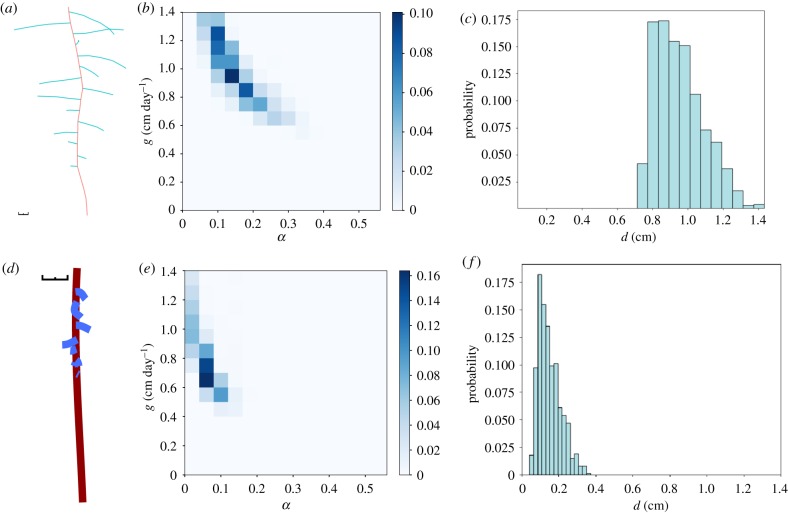
Posterior distributions on *Arabidopsis thaliana* and *Lupinus angustifolius* roots generated using RootBox. (*a*) Output from RootBox simulation of *Lupinus angustifolius* root growth; black scale bar is 1 cm. (*b*,*c*) Output posteriors from an ABC SMC framework run on RootBox output of *Lupinus angustifolius*, with final ABC SMC tolerance *ε* = 0.4 (Material and methods). (*b*) Two-dimensional posterior on primary root growth rate *g* and lateral root growth scaling *α*. (*c*) Posterior distribution on branch separation *d* in the RootBox growth model. (*d*) Output from RootBox simulation of *Arabidopsis thaliana* root growth; black scale bar is 1 cm. (*e*,*f*) Output posteriors from an ABC SMC framework run on RootBox output of *Arabidopsis thaliana*, with final ABC SMC tolerance *ε* = 0.4 (Material and methods). (*e*) Two-dimensional posterior on primary root growth rate *g* and lateral root growth scaling *α*. (*f*) Posterior distribution on branch separation *d* in the RootBox growth model. (Online version in colour.)

Figure 2 shows the resultant posteriors after applying our inference approach using RootBox as the core mechanistic model. Once more, the original generative parameters are well supported by the resulting posteriors, which also agree with the inferred values for primary and lateral growth rates using our simplified core model above (figure 1). The inter-lateral distances *d*, present in RootBox but not above, are also well recovered by the inference process. The maximum number of branches *b*_max_ is not tightly constrained by the synthetic data (electronic supplementary material, figure S2).

### 2.4. Inferring mechanistic parameters for wild-type *Arabidopsis thaliana* root systems

**Figure 3.**
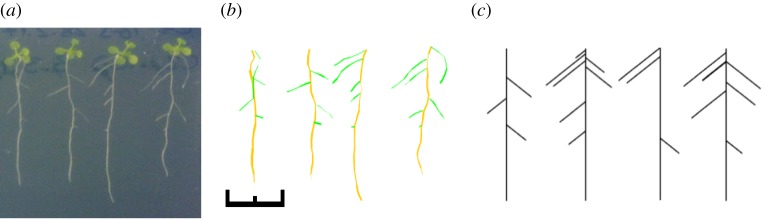
Example data and simulation output for the root-inference framework. (*a*) *Arabidopsis* seedlings grown vertically on agar (Material and methods) provide example root systems for the analysis pipeline. (*b*) Digitization of the root systems using SmartRoot [35] provides the quantitative data used in the inference process. (*c*) Example outputs from the stochastic growth model with parameters identified through the ABC SMC inference process. Black scale bar is 1 cm. (Online version in colour.)

To test the pipeline on experimental data, we grew *Arabidopsis* Col-0 plants on vertical (1/2) MS agar plates (Material and methods) and used a digital camera to capture their root system structure over several days. We used SmartRoot image analysis software [35] to extract the lengths and placements of tap and lateral roots from these digital images at each sampled timepoint. An example of the digitized data is shown in figure 3.

**Figure 4.**
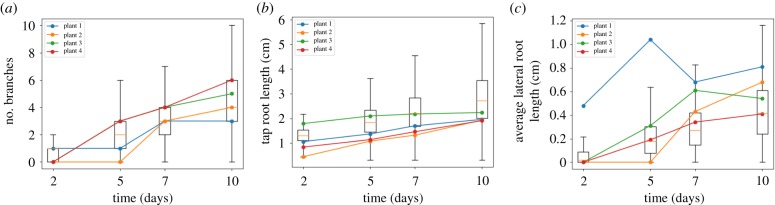
Summary statistic comparison between data and parametrized model. Individual line traces in each plot show time series of the summary statistic from observed *Arabidopsis* seedlings in figure 3; boxplots give the range of values arising from stochastic model simulation after parameter values have been learned. (*a*) Number of branch points; (*b*) primary root length; (*c*) average lateral root length. (Online version in colour.)

We applied our ABC SMC framework to estimate the posterior distributions of the mechanistic parameters underlying the development of these root systems. The earlier, high-*ε* populations of the SMC process gave a diverse range of simulated root structures; by the final population, the simulation outputs provide excellent visual matches to the observed experimental structures (figure 3) given the deliberate simplicity of the model. This intuitive snapshot matching is supported by the good agreement between the experimentally observed time series of summary statistics and those arising from simulation with the final posteriors (figure 4). Here, both the mean and the variability in the experimental statistics over time are captured by the distributions of simulated behaviour arising from the posteriors.

**Figure 5.**
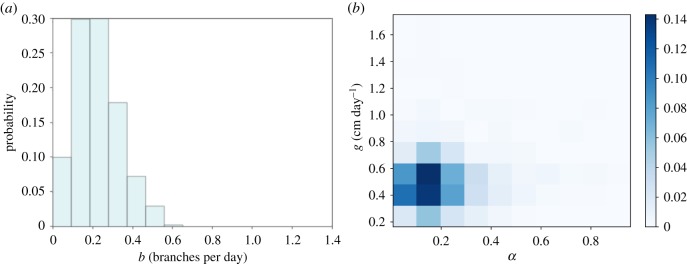
Posterior distributions on mechanistic parameters for *Arabidopsis* seedling roots. Posteriors from our ABC SMC framework run on the data from figure 3 with final tolerance *ε* = 2.5. (*a*) Posterior distribution on branching rate *b*. (*b*) Two-dimensional posterior on primary root growth rate *g* and lateral root growth scaling *α*. (Online version in colour.)

The posterior distributions themselves are shown in figure 5. The primary root growth rate *g* is reasonably well constrained, with a mean that intuitively falls around the total growth average. Notably, the posterior distribution on *g* is tighter than for the synthetic data example. This refinement reflects the strength of including time-course data in the inference platform. Observations of systems at different times provide more information on dynamic rate parameters, allowing better estimates than are available from single-instance observations alone.

The scaling of lateral growth rate *α* has a broader variance, reflecting the greater variability in average lateral root length observed in the data, and is correlated to some extent, as expected, with the value of *g*. The distribution of branching rate *b* is also broad, reflecting a greater variability in the experimental observation of branch number over time, and also the stochastic nature of this process: as *b* reflects the mean rate of a Poisson process, the same branching structure can be achieved with a variety of different *b* values. The modal value of *b* matches the average branching rate observed in the data. Overall, therefore, the ABC SMC framework gives reliable and intuitive readouts linked to both the average observed behaviour and plant-to-plant variability in the root structure.

### 2.5. Model selection for root growth and branching mechanisms

We next asked whether our approach could select between competing generative models, given time course data on the evolution of a root system. To this end, we considered a range of possible generative mechanisms for root growth and branching. We will employ uniform priors over competing models, reflecting the fact that, before any observations are made, we have no belief that one mechanism is more likely than another. This prior belief can of course be arbitrarily changed within our Bayesian framework to reflect prior information. We then use our ABC SMC framework to identify the posterior support for each mechanistic model, given the observed data [33] (Material and methods).

First, we consider different elongation laws for root growth. The first model involves root growth at a constant rate; the second involves a negative-exponential growth law supported by [29] of the form

**Figure 6.**
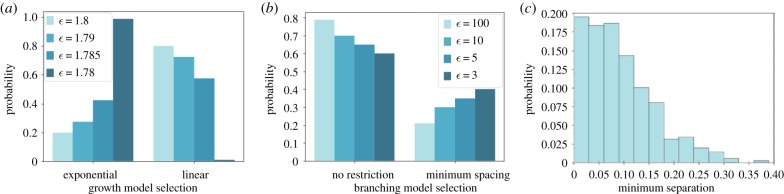
ABC SMC allows selection of competing mechanistic models for root growth. (*a*) Model selection posteriors from comparing a simple, constant-rate growth model to the negative-exponential model used in CRootBox [29] for decreasing ABC tolerance *ε*. The negative-exponential model acquires greater support as the tolerance decreases. (*b*) Model selection posteriors from comparing a uniform branching model to a model imposing a minimum separation from existing branches. The more parsimonious uniform model experiences higher support with relaxed tolerance but the minimum-separation model gains support with tighter tolerances (tolerances are higher for this model selection run, reflecting a tradeoff with the greater computational resource required). (*c*) Posterior for the minimum branch separation, *δ*, required when the minimum spacing model is implemented. (Online version in colour.)

2.3 $$l(t)=l_{\max}(1-{\text{e}}^{-gt/l_{\max}}),$$

where *l*(*t*) is the length of the root at time *t*, parametrized by a rate constant *g* and scaling constant *l*_max_. The posterior distribution over model index through the SMC process is shown in figure 6*a*,*b*. The most permissive population (highest *ε*) shows less support for the exponential model, favouring model parsimony. As a better fit to the data is required, the support for the exponential model increases until it overcomes the lower weightings due to the additional parameter and is preferentially selected.

Next, we explore a more nuanced mechanistic question underlying root architecture. We compared two models for branch placement positions. First, a uniform branching model, where the branching location was chosen at random anywhere along the primary root. Second, a minimally spaced model, which imposed a distance parameter *δ* around each existing branch where no further branching could occur. If a branch was attempted within this distance no branch was implemented and the algorithm continues. Figure 6*b*,*c* shows the model selection posteriors with decreasing tolerance, and the posterior on *δ* when the minimally spaced model was implemented. Here, the posteriors for the spacing model are lower for the more permissive populations, reflecting the increased model complexity—the extra parameter *δ* makes the model less parsimonious. The support for the model increases as a better fit to data is required in the subsequent populations. By the final tolerance, the models have comparable support. Hence, the dataset suggests roughly equal support for both models despite their difference in complexity.

These simple experiments serve to illustrate the ability of ABC SMC to provide statistical support for competing mechanistic hypotheses (for example, linear versus negative-exponential root elongation laws). There is, however, nothing to prevent other targeted mechanistic questions being addressed using this framework (see Discussion).

### 2.6. Comparison between root structures

Next, we asked whether the ABC SMC framework could distinguish between two phenotypes—those corresponding to wild-type *Arabidopsis* and the *friendly* mutant line. *FRIENDLY* is a mitochondrial fusion gene that when compromised has a range of bioenergetic effects which lead to reduced root growth [36].

**Figure 7.**
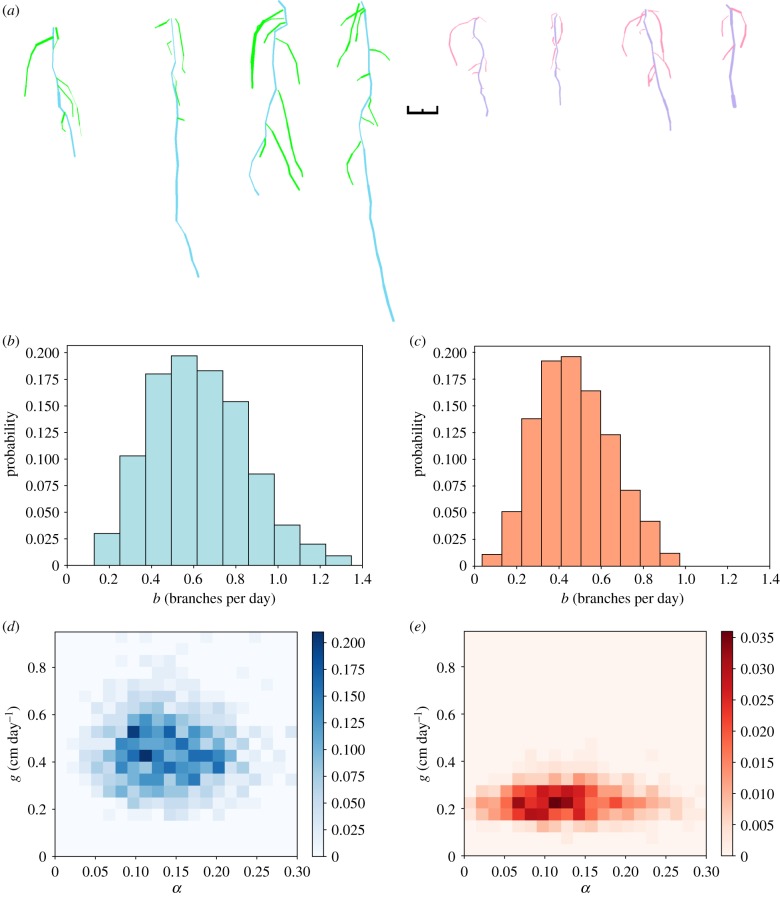
Distinguishing phenotypes with mechanistic inference. (*a*) (left) Wild-type and (right) *friendly* *Arabidopsis* seedlings grown in agar as described in §4.1, demonstrating the root growth phenotype of *friendly*. The tracings were produced using SmartRoot software [35] and the colours adjusted; black scale bar is 1 cm. (*b*,*c*) Output posteriors for branching rate *b* show similar distributions for wild-type and *friendly*. (*d*,*e*) Two-dimensional posteriors on primary root growth rate *g* and lateral root growth scaling *α* demonstrate clear separation, reflecting the reduced root growth observed in *friendly*. (Online version in colour.)

Wild-type and *friendly* plants were grown under the same conditions as above (Material and methods), and the inference pipeline was run as before, with exponential growth and uniform branching. The output posteriors in figure 7 reflect the differing root systems shown in the tracings, with a clear separation in the parameter space between the two phenotypes. The branching rate *b* is fairly unconstrained as observed in §2.4 due to inherent stochasticity in the branching mechanism. The values of *g* vary significantly between wild-type and *friendly*, as reflected in the tracings, with little change in the value of *α*. The distribution of *g* is substantially shifted towards lower values for the *friendly* plants, reflecting the known challenge to root growth resulting from this mutation. *l*_max_ shows a wide variability in both phenotypes, while representing clear differences consistent with the reduced root growth observed in the *friendly* mutant line. There is a very little constraint in the value of *l*_max_, suggesting little reliance on the value, although smaller values appear to be favoured for the *friendly* phenotype.

Taken together, these results demonstrate that the physical parameters governing root architecture growth can be learned using this ABC SMC approach, and uncertainty in these learned outcomes quantified. The mechanistic model within our inference process both allows us to harness time-course data and dissects which parameters change (here, growth rate *g*) and which remain similar (here, lateral root scaling *α*) in different cases. This is a key strength of this method and would be difficult to obtain through traditional parameter inference methods. The platform readily identifies the physical different mechanisms underlying root architecture in a mutant line and identifies accepted physical model structures for root growth.

## 3. Discussion

We have presented a framework for the inference of parameter values and mechanisms in root growth models when applied to an observed root system. While there has been much work undertaken producing plant root simulations from given parameters, our approach addressed the much less-studied ‘inverse problem’: that of finding generative parameter values and mechanisms that can reproduce a given root system. Knowledge about these mechanisms and parameters, and their ranges, flexibility and relative importance, is necessary for an understanding of root growth processes such as growth and branching decisions, and how these may relate to biological processes within the plant. We hope that this highly general approach will allow for a more mechanistic understanding of root growth and to quantify the efficacy of existing models.

We first used a very general growth model to (i) retain consistency with the ‘core’ of the maximum possible number of existing growth models and (ii) focus on parameters related to the growing plant and its phenotype, rather than the specifics of its physical embedding. We have demonstrated using RootBox [28] that our approach can readily be adapted to other specific existing root models to allow the quantification of values of and uncertainty in generative parameters, furthering understanding of root system architecture. We also illustrated how alternative hypothesized mechanistic models can straightforwardly be compared, using SMC model selection. A strength of the Bayesian embedding here is that the most parsimonious model that is capable of explaining observations is naturally selected in the case of models with different numbers of parameters [33].

Advances in imaging techniques are allowing for greater insight into root system architecture [8] and specially designed image analysis software [37] allows for increasingly efficient data collection from images. The combination of root models, advanced imaging techniques, image analysis software and an SMC framework could allow further advances in our understanding of root growth. We anticipate that, with the increasing developments in root imaging, this technique will find application in a growing variety of datasets, allowing for the investigation of generative parameters for a wide variety of root system phenotypes. A natural future extension for this work would be to perform inference based directly on image data, rather than statistics of these data. This approach would require simulation of the imaging process as well as the generation of model root systems, for example, embedding the idealized root system in a simulated soil substrate and simulating the artefacts and noise involved in the imaging process. While (much) more computationally intensive, this approach would allow a more direct leveraging of phenotype data from experimental studies.

Notably, our approach allows inference based on time-course measurements of a developing root system, which increase the power and precision with which parameters and mechanisms can be identified. As demonstrated with our synthetic examples, this approach can readily be applied to single-instance observations but also naturally leverages dynamic information to refine posterior distributions on physical rate parameters.

A stochastic modelling framework for root growth allows for a wide variety of possible outputs to be considered in the inference process, reflecting the variation between root systems in the real world. In this way, the modelling approach allows for investigation into the underlying mechanisms which are widely applicable, while avoiding a reliance on specificities and overfitting to a particular phenotype or growth environment. Predicting a branching event would require the consideration of processes such as genetics, cellular interactions and organism-scale resource partitioning [38], necessitating the development of a multiscale framework. As such multiscale approaches develop, we anticipate the use of likelihood-free inference to be further embraced to resolve inverse problems in parameter identification.

While the generality of our approach is appropriate for the scope of this study, greater specificity is required to gain a true understanding of plant processes. Care needs to be taken in the application of ABC techniques: choices must be made over elements such as the tolerance, priors and summary statistics to achieve a balance between convergence rate and specificity of results. As specific choices for these values can be hard to interpret, simulation outputs must be verified to provide a reasonable match to genuine behaviour (as we have attempted throughout). We have worked with different models to explore the behaviour of our method under different generative assumptions. In Bayesian model selection, prior beliefs about models can strongly affect their support and interpretation must take this into account [33]. However, we have aimed to demonstrate the strength of this approach when carefully applied and interpreted.

Overall, we have demonstrated a technique to allow for greater insight into model parameters for root systems, which could aid in increasing understanding of root growth mechanisms. The generalized approach allowed for investigation of the key aspects underlying root topology while being highly adaptable for use with existing root architecture models.

## 4. Material and methods

### 4.1. Plant growth

*Arabidopsis* Col-0 and *friendly* seeds were sterilized with three 3 min washing steps in 50% domestic bleach and water rinses, then plated on (1/2) MS agar in vertical plates. Plants were grown at constant 25°C on a 16 h light/8 h dark cycle. Plates were photographed over a time course of 2, 5, 7 and 10 days to produce time-series images of the seedling growth. Summary statistics were extracted from the images using SmartRoot [35], an imageJ plugin. The root systems were traced manually using thresholding, producing a skeleton over the original image. Summary statistics on root length and branch placement were then recorded from this skeleton.

### 4.2. Model structure

Root growth was simulated using a hybrid stochastic–deterministic algorithm. Primary root growth, by default, was assumed to follow a negative-exponential growth law

4.1 $$l(t)=l_{\max}(1-{\text{e}}^{-gt/l_{\max}}),$$

parametrized by a rate constant *g* and a scaling constant *l*_max_. The alternative uniform growth model simply took the form *l*(*t*) = *gt*. Lateral roots grow according to the same growth law as the primary root, but with a multiplicative factor *α* applied to *g* so that for lateral roots *g*_*l*_ = *αg*.

Branching was treated as a Poisson event with rate parameter *b*. The time until the next branching event is found using the Gillespie algorithm [39], and the length of existing branches is updated from the current time until the time of the branching event. The branching location was then determined by a specified branching model, initially specified as a uniform probability distribution along the length of the primary root. In visualizing structures, branching angle was always set to an angle of 45° from the growth direction, with the equal change of being placed each side of the primary root, although these angles and positions play no role in the simulation. These steps are repeated until the time of the next branch exceeds the maximum simulation time, at which point the branch lengths are updated up to the maximum simulation time, and no branching event occurs. Once a branching event has occurred, the sidebranch grows according to the same growth law as the main root, scaled by parameter *α*; variability in lateral root length thus corresponds to variability in initial branching times and positions.

### 4.3. ABC SMC implementation

An ABC framework was implemented in Matlab. Model parameters were drawn from specified distributions and passed to the model as described in model structure above. Broadly, the simulated root systems are then compared to data, and the parameter values accepted if the simulation is sufficiently close to the data, with tolerance *ε* defined at the time of implementation. If the previous values were accepted, the parameter values are perturbed with a perturbation kernel *K*_*t*_. If the previous values were not accepted, the parameter values were drawn from the priors as previously described. This process was repeated until 1000 hits were obtained at the specified tolerance.

We follow [33] in our ABC SMC implementation. For completeness, algorithm 2 introduces a simple rejection-sampling scheme under ABC. Algorithm 2 embeds this scheme in an SMC framework for parameter inference and model selection.

**Algorithm 1.** ABC rejection sampling for parameter inference.

(1) Given $N_{\;p}$ plant structures and $N_{t}(i)$ longitudinal observations for plant i, characterize the summary statistics $d_{ij}=\{B,\;L,\;\hat{l}\}$ from every plant i and observation j in the dataset. (2) Draw a trial set of parameters $\theta^{*}$ from the prior distribution π(θ). (3) Simulate $N_{\;p}$ instances of root growth, recording the state of structure i at each of the $N_{t}(i)$ time points corresponding to an experimental observation. (4) Compute ρ using equation (2.2) above, to give the separation between each recorded structure and its simulated counterpart. (5) If ρ<ϵ, where ϵ is a given tolerance, accept $\theta^{*}$ as a sample from the posterior. (6) If a termination condition is not met, return to 2.

**Algorithm 2.** ABC SMC for parameter inference and model selection.

(1) Given $N_{\;p}$ plant structures and $N_{t}(i)$ longitudinal observations for plant i, characterize the summary statistics $d_{ij}=\{B,\;L,\;\hat{l}\}$ from every plant i and observation j in the dataset. (2) Initialize tolerance vector E containing T elements. Set population indicator t=0. (3) Set particle indicator i=1. (4) Sample model indicator $m^{*}$ from prior π(m). (5) If t=0, sample $\theta^{**}$ from $\pi (\theta (m^{*}))$. If t>0, sample $\theta^{*}$ from the previous population $\left\{\theta {\left(m^{*}\right)}_{t-1}\right\}$ with weights $w{\left(m^{*}\right)}_{t-1}$, and set $\theta^{**}∼K_{t}(\theta |\theta^{*})$. (6) If $\pi (\theta^{**})=0$, go to 4. (7) Simulate $N_{\;p}$ instances of root growth using $\theta^{**}$, recording the state of structure i at each of the $N_{t}(i)$ time points corresponding to an experimental observation. (8) Compute ρ using equation (2.2) above. (9) If ρ≥E[t], go to 4. (10) Set $m_{t}^{\left(i\right)}=m^{*}$ and add $\theta^{**}$ to the population $\left\{\theta {\left(m^{*}\right)}_{t}\right\}$. If t=0, set weights $w_{t}^{\left(i\right)}=0$, otherwise $$w_{t}^{\left(i\right)}=\frac{\pi (\theta^{**})}{\sum_{\;j=1}^{N}w_{t-1}^{\left(\;j\right)}K_{t}(\theta_{t-1}^{\left(\;j\right)},\theta^{**})}.$$ If i<N, set i=i+1, go to 4. (11) For every m, normalize the weights. If t<T, set t=t+1, go to 3.

For a single model, the prior *π*(*m*) associated with that model is unity and the choice of model indicator m* plays no role in the inference process.

We used uniform priors over all model structures for *π*(*m*) and uniform priors between 0−1.4 day^−1^ for *g*, 0−1.4 day^−1^ for *b*, 0–1 for *α* and 5–40 cm for *l*_max_. The perturbation kernel we used was *K*_*t*_ ∼ *N*(0, 0.1*P*), where *P* is the width of the uniform prior. The tolerance vector was **E** = *ε*{5, 3, 2, 1.5, 1}.

## Supplementary Material

Supplementary Figure 1

## Supplementary Material

Supplementary Figure 2

## Data accessibility

The data and code used are freely available in Github repository https://github.com/StochasticBiology/root-inference.
